# Dipeptidyl Peptidase (DPP)-4 Inhibitors and Pituitary Adenylate Cyclase-Activating Polypeptide, a DPP-4 Substrate, Extend Neurite Outgrowth of Mouse Dorsal Root Ganglia Neurons: A Promising Approach in Diabetic Polyneuropathy Treatment

**DOI:** 10.3390/ijms25168881

**Published:** 2024-08-15

**Authors:** Masahiro Yamaguchi, Saeko Noda-Asano, Rieko Inoue, Tatsuhito Himeno, Mikio Motegi, Tomohide Hayami, Hiromi Nakai-Shimoda, Ayumi Kono, Sachiko Sasajima, Emiri Miura-Yura, Yoshiaki Morishita, Masaki Kondo, Shin Tsunekawa, Yoshiro Kato, Koichi Kato, Keiko Naruse, Jiro Nakamura, Hideki Kamiya

**Affiliations:** 1 Division of Diabetes, Department of Internal Medicine, Aichi Medical University School of Medicine, Nagakute 480-1185, Japan; 2 Department of Innovative Diabetes Therapy, Aichi Medical University School of Medicine, Nagakute 480-1185, Japan; 3 Department of Medicine, Aichi Gakuin University School of Pharmacy, Nagoya 464-8650, Japan; 4 Department of Internal Medicine, Aichi Gakuin University School of Dentistry, Nagoya 464-0821, Japan

**Keywords:** diabetic polyneuropathy, dipeptidylpeptidase-4, pituitary adenylate cyclase-activating polypeptide, neurite outgrowth of dorsal root ganglia neurons

## Abstract

Individuals suffering from diabetic polyneuropathy (DPN) experience debilitating symptoms such as pain, paranesthesia, and sensory disturbances, prompting a quest for effective treatments. Dipeptidyl-peptidase (DPP)-4 inhibitors, recognized for their potential in ameliorating DPN, have sparked interest, yet the precise mechanism underlying their neurotrophic impact on the peripheral nerve system (PNS) remains elusive. Our study delves into the neurotrophic effects of DPP-4 inhibitors, including Diprotin A, linagliptin, and sitagliptin, alongside pituitary adenylate cyclase-activating polypeptide (PACAP), Neuropeptide Y (NPY), and Stromal cell-derived factor (SDF)-1a—known DPP-4 substrates with neurotrophic properties. Utilizing primary culture dorsal root ganglia (DRG) neurons, we meticulously evaluated neurite outgrowth in response to these agents. Remarkably, all DPP-4 inhibitors and PACAP demonstrated a significant elongation of neurite length in DRG neurons (PACAP 0.1 μM: 2221 ± 466 μm, control: 1379 ± 420, *p* < 0.0001), underscoring their potential in nerve regeneration. Conversely, NPY and SDF-1a failed to induce neurite elongation, accentuating the unique neurotrophic properties of DPP-4 inhibition and PACAP. Our findings suggest that the upregulation of PACAP, facilitated by DPP-4 inhibition, plays a pivotal role in promoting neurite elongation within the PNS, presenting a promising avenue for the development of novel DPN therapies with enhanced neurodegenerative capabilities.

## 1. Introduction

Diabetic polyneuropathy (DPN), a most relevant complication of diabetes, manifests symmetrically in a nerve length-dependent manner, marked by the degeneration of both myelinated and unmyelinated fibers in a dying-back pattern [1,2,3,4,5]. DPN’s clinical landscape is defined by an array of distressing symptoms, including pain, paresthesia, and sensory deficits, and DPN affects approximately 50% of people with considerable morbidity, mortality, and diminished quality of life. Several reports have demonstrated that histopathological findings indicative of neuropathies similar to those in humans have been observed in diabetic model mice [6,7,8]. Through these animal models, it has been established that polyol, hexosamine, protein kinase C, advanced glycation end-product pathways, and oxidative stress are implicated in neurodegeneration in the context of hyperglycemia [9,10,11,12,13,14,15,16,17]. However, despite the accumulation of this evidence, a definitive therapeutic approach remains elusive. Therefore, the imperative for identifying novel therapeutic targets centered on neuronal protection has become paramount in the comprehensive management of DPN, necessitating a paradigm shift towards innovative strategies for effective intervention and amelioration of its deleterious impact on affected individuals.

Incretin hormones, including Glucagon-like peptide (GLP)-1 and Glucose-dependent insulinotropic polypeptide, constitute a crucial family of peptides that play a pivotal role in potentiating insulin secretion post-enteral nutrient ingestion [18,19,20]. Despite their therapeutic promise, however, their efficacy is hindered by rapid enzymatic inactivation in vivo by dipeptidyl-peptidase (DPP)-4 or CD26, leading to the formation of biologically inactive metabolites. The advent of DPP-4 inhibitors, which elevate circulating incretin levels by impeding enzymatic degradation, has emerged as a groundbreaking strategy that has demonstrated utility in managing both diabetic patients and experimental models of type 2 diabetes [21,22,23]. Our research, therefore, delves into elucidating the diverse biological impacts of DPP-4 inhibitors, specifically within the peripheral nervous system (PNS). Numerous studies have highlighted the multifaceted effects of DPP-4 inhibitors, extending beyond glycemic control to encompass broader physiological processes such as the regulation of inflammation within adipose tissues, mediated by a spectrum of DPP-4 substrates encompassing chemokines and neuropeptides [24,25,26,27,28,29,30]. Within this milieu of DPP-4 substrates, a notable subset comprises neurotrophic factors [31,32,33,34,35,36,37], each bearing distinct yet interconnected roles in neurobiology and metabolic regulation. For instance, pituitary adenylate cyclase-activating polypeptide (PACAP) emerges as a multifunctional entity functioning as a hypothalamic hormone, neurotransmitter, and neurotrophic factor crucial for neuronal survival and plasticity [38]. Likewise, Neuropeptide Y (NPY) assumes a central position in modulating diverse physiological functions spanning from food intake and energy homeostasis to circadian rhythm regulation and cognitive processes [39]. Furthermore, the influence of stromal cell-derived factor (SDF)-1α, a neural chemokine, extends to modulating neurite outgrowth, underscoring its significance in neural development and repair processes [40,41]. In addition to their well-documented impact on metabolic parameters, recent studies have underscored the potential of DPP-4 inhibitors in mitigating the development of DPN, particularly evident in type 1 diabetic rodent models [42,43,44,45]. This growing body of evidence not only reinforces the therapeutic relevance of DPP-4 inhibitors but also underscores the intricate interplay between metabolic signaling pathways and neurological outcomes, offering a nuanced perspective on the comprehensive impact of these agents in diabetes management and beyond.

Here, we advanced the hypothesis that DPP-4 inhibitors, renowned for their regulatory role in incretin degradation, extend their influence to encompass not only incretins but also a spectrum of chemokines and neuropeptides, thereby manifesting neurotrophic effects. Through meticulous experimentation and rigorous analysis conducted in this study, we embarked on the validation process to ascertain the direct impact of DPP-4 inhibitors and/or DPP-4 substrates on the PNS. Our pursuit aimed to unravel the intricate mechanisms underpinning the neurotrophic potential of these inhibitors, delving into their capacity to modulate pathways critical for neuronal health and function. By elucidating the intricate interplay between DPP-4 inhibitors, substrates, and PNS dynamics, our findings promise to contribute significantly to the burgeoning field of neuropharmacology, shedding light on novel avenues for therapeutic interventions targeting neurological disorders. This exploration not only enriches our understanding of DPP-4 inhibitor mechanisms but also underscores their broader implications in fostering neural resilience and combating pathological conditions affecting the nervous system.

## 2. Results

### 2.1. DPP-4 Expresses in Dorsal Root Ganglia (DRG)

**Figure 1:**
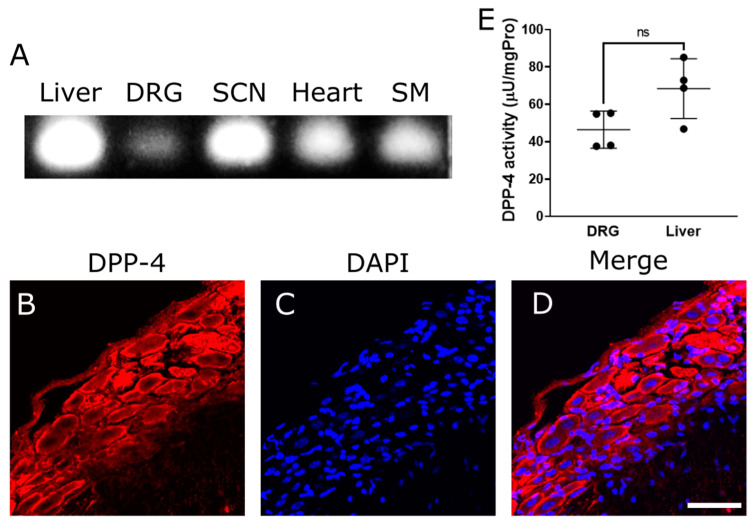
Expression and activity of DPP-4 in the peripheral nervous system. (**A**) RT-PCR of DPP-4 mRNA on several tissues: liver, dorsal root ganglia (DRG), sciatic nerve (SCN), heart, and skeletal muscle (SM). (**B**–**D**) Immunostaining of DPP-4 protein on DRG. (**B**): DPP-4. (**C**): DAPI. (**D**): Merge. Scale bar: 50 μm. (**E**) Quantified DPP-4 activity of DRG and liver, μU: microunits; mgPro: mg Protein; ns: not significant between DRG and liver.

In our investigation aimed at confirming the presence of DPP-4 expression within the PNS, specifically targeting the sciatic nerve and DRG, we employed a combination of reverse transcription-polymerase chain reaction (RT-PCR) and immunostaining techniques. As a result, the findings from the RT-PCR analysis unequivocally demonstrated the presence of DPP-4 mRNA within both the sciatic nerve and DRG (Figure 1A). Furthermore, our immunostaining assays revealed an expression of DPP-4 on DRG neurons (Figure 1B–D). Rónai et al. showed that DPP-4 activity is present in rat lumbar DRG [46]. Therefore, we investigated whether mouse DRG exhibits DPP-4 activity. Our DPP-4 activity assays revealed that DRG has activity levels comparable to those of the liver (Figure 1E). These compelling results not only validate the expression of DPP-4 in crucial components of the PNS but also shed light on its potential role within the DRG microenvironment. These results suggest that DPP-4 inhibitors directly act on DRG.

### 2.2. DPP-4 Inhibitors Promoted Neurite Outgrowth of DRG Primary Neurons

**Figure 2:**
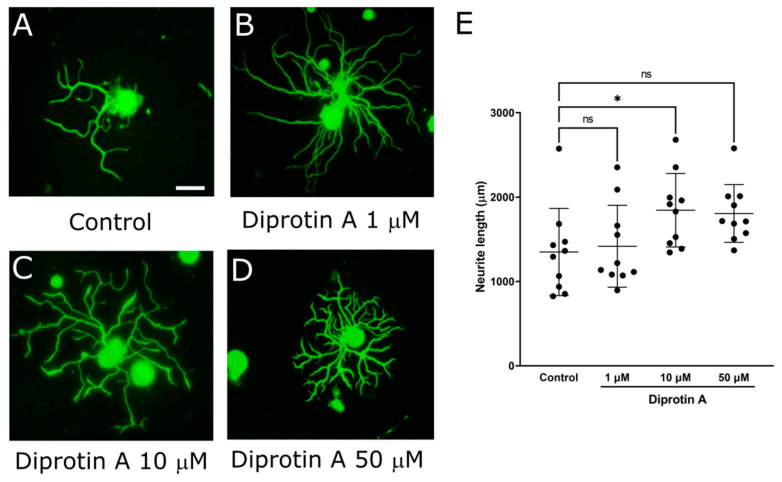
Neurite outgrowth of DRG neurons by Diprotin A. (**A**–**D**) Representative fluorescence figures of DRG neurons cultured in the absence (**A**) or presence (**B**–**D**) of Diprotin A ((**B**): 1 μM, (**C**): 10 μM, (**D**): 50 μM). Scale bar: 50 μm. (**E**) Quantified neurite length in each DRG neuron. *: *p* < 0.05 versus control; ns: not significant versus control.

**Figure 3:**
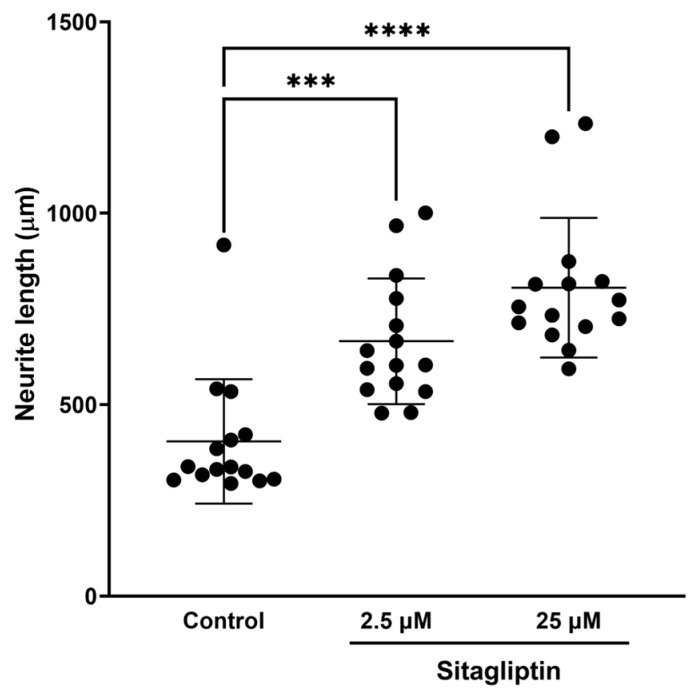
Quantified neurite length in each DRG neuron with or without sitagliptin. ***: *p* < 0.001, ****: *p* < 0.0001 versus control.

**Figure 4:**
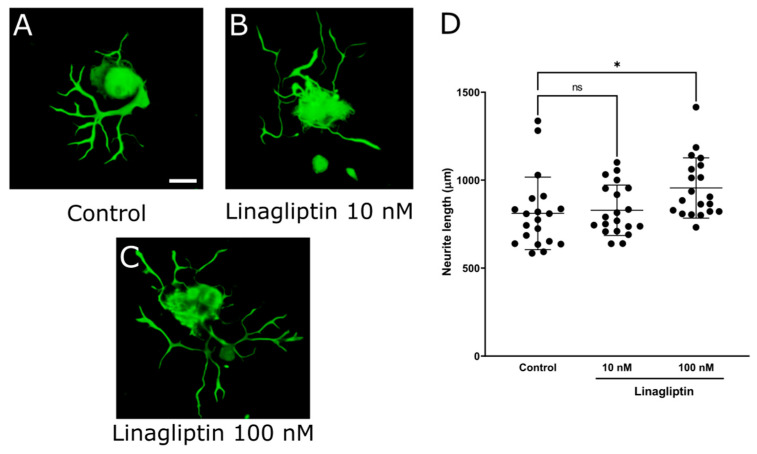
Neurite outgrowth of DRG neurons by linagliptin. (**A**–**C**) Representative fluorescence figures of DRG neurons cultured in the absence (**A**) or presence (**B**,**C**) of linagliptin ((**B**): 10 nM, (**C**): 100 nM). Scale bar: 50 μm. (**D**) Quantified neurite length in each DRG neuron. *: *p* < 0.05; ns: not significant versus control.

It is considered that DPP-4 inhibitors affect neurite outgrowth of DRG neurons because they protect imperception and reduce intraepidermal nerve fiber density (IENFDs) in type 1 diabetic rodent models [42,44,45]. To evaluate the impact of various DPP-4 inhibitors (Diprotin A, sitagliptin, and linagliptin) on DRG neurons, we assessed the neurite outgrowth of DRG neurons using our DRG primary culture system. First, the mean neurite length in each DRG neuron was significantly increased by Diprotin A (Figure 2; control: 1349 ± 517 μm, Diprotin A 10 μM: 1845 ± 436, *p* < 0.05). Second, the mean neurite length in each DRG neuron was significantly increased by sitagliptin (Figure 3; control: 404 ± 163 μm, sitagliptin 25 μM: 806 ± 182, *p* < 0.0001). Finally, the mean neurite length in each DRG neuron was significantly increased by linagliptin (Figure 4; control: 811 ± 206 μm, linagliptin 100 nM: 956 ± 171, *p* < 0.05). These compelling results strongly suggest that DPP-4 inhibitors play a pivotal role in elongating the neurite length of DRG neurons by inhibiting the degradation of DPP-4 substrates.

### 2.3. PACAP, DPP-4 Substrate, Promoted Neurite Outgrowth of DRG Primary Neurons

**Figure 5:**
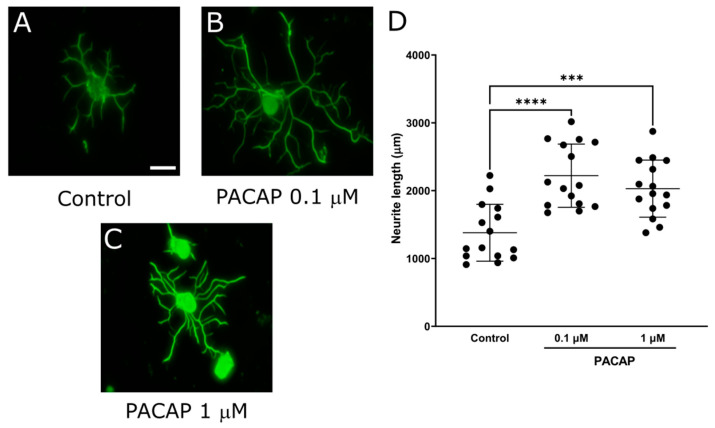
Neurite outgrowth of DRG neurons by PACAP. (**A**–**C**) Representative fluorescence figures of DRG neurons cultured in the absence (**A**) or presence (**B**,**C**) of PACAP ((**B**): 0.1 μM, (**C**): 1 μM). Scale bar: 50 μm. (**D**) Quantified neurite length in each DRG neuron. ***: *p* < 0.001, ****: *p* < 0.0001 versus control.

**Figure 6:**
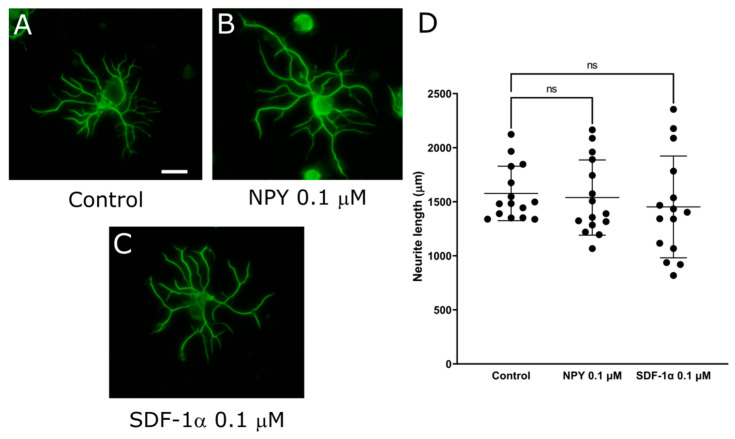
Neurite outgrowth of DRG neurons by NPY and SDF-1α. (**A**–**C**) Representative fluorescence figures of DRG neurons cultured in the absence (**A**) or presence of (**B**) NPY 0.1 μM and (**C**) SDF-1α 0.1 μM. Scale bar: 50 μm. (**D**) Quantified neurite length in each DRG neuron. ns: not significant versus control.

Due to the results that DPP-4 inhibitors promoted neurite outgrowth, we next focused on DPP-4 substrates. Since PACAP, NPY, and SDF-1α are known as neurotrophic factors, we assessed the neurite outgrowth of DRG neurons to evaluate their impact on DRG neurons. The mean neurite length in each DRG neuron was significantly increased by PACAP (Figure 5; control: 1379 ± 420 μm, PACAP 0.1 μM: 2221 ± 466, *p* < 0.0001). The supplementation of NPY or SDF-1α showed no significant change in the neurite length (Figure 6; control: 1577 ± 252 μm vs NPY0.1 μM: 1538 ± 348, SDF-1α 0.1 μM: 1452 ± 471). These results indicated that PACAP might elongate the neurite length of DRG neurons, thus highlighting its potential as a key player of DPP-4 substrate in neurotrophic modulation.

### 2.4. DPP-4 Inhibitors and PACAP Enhanced Cell Viability in 50B11 Cells, DRG Neuronal Cells

**Figure 7:**
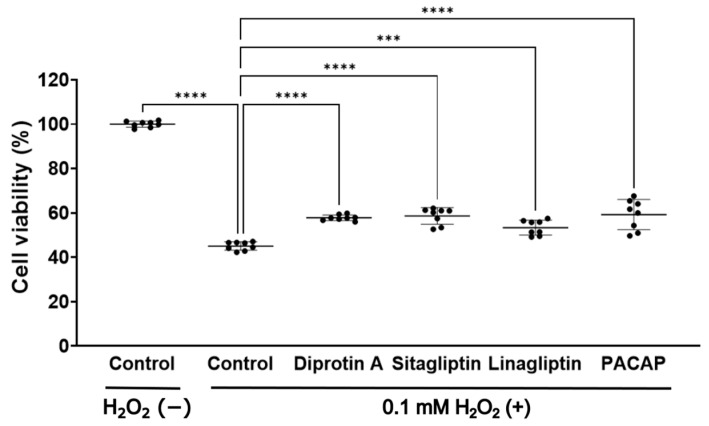
Cell viability in 50B11 cells treated with DPP-4 inhibitors and PACAP. H_2_O_2_ (−): no supplementation of H_2_O_2_. 0.1 mM H_2_O_2_ (+): supplementation of 0.1 mM H_2_O_2_. 100% cell viability: control without H_2_O_2_. H_2_O_2_: hydrogen peroxide. ***: *p* < 0.001, ****: *p* < 0.0001 versus control with 0.1 mM H_2_O_2_.

Considering the neurotrophic effects of DPP-4 inhibitors and PACAP, we investigated their mechanisms of action. Our previous studies have highlighted the neuroprotective and neurite outgrowth-promoting effects of glucagon and glucagon-like peptide-1 (GLP-1) analogs using the 50B11 cell line, an immortalized cell line of rat DRG neurons [47,48,49,50]. Given that DPP-4 inhibitors elevate GLP-1 and PACAP levels, we speculated that they may exhibit neuroprotective effects through these peptides. We evaluated their neuroprotective effects against the cytotoxicity induced by hydrogen peroxide (H_2_O_2_) in 50B11 cells using 3-(4,5-dimethylthiazol-2-yl)-5-(3-carboxymethoxyphenyl)-2-(4-sulfophenyl)-2H-tetrazolium bromide (MTT) assay. Our MTT assay revealed that DPP-4 inhibitors and PACAP inhibited the decrease in cell viability (Figure 7). This result suggests that DPP-4 inhibitors and PACAP enhanced cell viability against H_2_O_2_.

## 3. Discussion

In this study, we used our DRG primary culture system to elucidate the mechanisms underlying neurotrophic effects mediated by DPP-4 inhibitors. First, our investigations demonstrated the presence of DPP-4 expression within DRG, laying a foundational understanding for subsequent experiments. Second, through rigorous experimentation, we unequivocally established that DPP-4 inhibitors play a pivotal role in extending neurite outgrowth of DRG neurons, illuminating a direct link between DPP-4 activity and neurotrophic responses. Third, our findings revealed a pronounced effect of PACAP on elongating neurite outgrowth, further delineating the multifaceted pathways involved in neurotrophic modulation. Meanwhile, our results unveiled that neither NPY nor SDF-1α contributed significantly to neurite outgrowth within DRG neurons.

In our previous report, we demonstrated the beneficial impact of Exendin-4, GLP-1 analog, on DPN in diabetic mice [51], complementing the existing literature that has shown that DPP-4 inhibitors mitigate the progression of DPN in rodent models of diabetes [42,43,44,45]. Expanding upon these findings, our current study has unveiled the expression of DPP-4 in DRG, and notably, we have discovered that DPP-4 inhibitors promote the elongation of neurites in DRG neurons independently of their hypoglycemic effects. This novel observation signifies a direct modulatory role of DPP-4 inhibitors within the DRG microenvironment. Our investigation marks the inaugural documentation of DPP-4 inhibitors directly facilitating the extension of neurites in DRG neurons, a phenomenon that, aside from its glycemic regulatory functions, holds promise in ameliorating the onset and progression of DPN through an alternative mechanism. This newfound understanding not only broadens our comprehension of DPP-4 inhibitor actions but also underscores the multifaceted nature of therapeutic interventions targeting peripheral neuropathies in the diabetic milieu, paving the way for nuanced approaches aimed at mitigating neuropathic complications beyond conventional glycemic control strategies.

In our investigation into the multifaceted neurotrophic effects of DPP-4 inhibitors on the PNS, we specifically directed our attention to three key neurotrophic factors among DPP-4 substrates. Our findings revealed a significant promotion of neurite outgrowth in DRG neurons by PACAP. This observation aligns with established knowledge linking PACAP to the differentiation of DRG neurons [52] and its heightened expression during pain induction within DRG [53]. Mechanistically, PACAP operates through the adenosine 3′, 5′-cyclic monophosphate (cAMP)-cAMP-dependent protein kinase A (PKA) signaling cascade via binding to the PAC1 receptor [54,55], akin to the actions of forskolin, a known cAMP activator that also extends neurite outgrowth in DRG neurons. Notably, PACAP belongs to the VIP/secretin/glucagon superfamily of peptides, further underscoring its relevance to neuronal function. Our previous work has also implicated the neuroprotective effects of glucagon gene-derived peptides, including glucagon and GLP-1, on the PNS of mice [56], thereby reinforcing the notion that PACAP likely confers neuroprotective benefits to the PNS as well. Although it is widely acknowledged in scientific circles that PACAP triggers the activation of phospholipase C-Ca^2+^ signaling pathways, there is a prevalent hypothesis suggesting a direct correlation between this signaling cascade and the augmentation of pain perception. This hypothesis is rooted in the understanding that heightened levels of PACAP can potentially aggravate pain sensations by acting as sensory transmitters that relay signals to the spinal cord [53]. Despite these concerns, the existing body of literature does not present any documented cases wherein DPP-4 inhibitors, which are known to elevate PACAP levels, contribute to the development or exacerbation of pain symptoms. This discrepancy prompts a closer examination of the localized effects of DPP-4 inhibition within the DRG microenvironment, especially considering the limited permeability of DPP-4 inhibitors across the blood-brain barrier. Experimental findings from our study, employing high-performance liquid chromatography (HPLC) to measure PACAP concentrations in DRG primary neuron media treated with DPP-4 inhibitors, revealed values at the detection limit. In the primary culture models, the neurotrophic responses elicited by PACAP showcased a bimodal pattern: when co-cultured with neurons and glial cells, effective concentrations ranged from 10^−13^ to 10^−11^ M, whereas for neurons alone, the range shifted to 10^−9^ to 10^−7^ M [57,58,59]. Given the complexity of the DRG primary culture system, encompassing both neurons and glial cells, it is plausible to infer that the upregulation of PACAP induced by DPP-4 inhibitors might exert neurotrophic effects even at exceedingly low concentrations, as indicated by our HPLC detection limits. These insights underscore the intricate interplay between PACAP signaling, DPP-4 inhibition, and neurotrophic responses within the microenvironment of DRG, highlighting avenues for further investigation into the nuanced roles of these signaling pathways in neuronal function. Although initially recognized as neurotrophic factors, both NPY and SDF-1a have not demonstrated their expected effects. There are some possibilities that NPY exhibits inhibitory action via binding to its Gi/o-coupled proteins [60], and SDF-1α, chemokine, does not exert an anti-inflammation effect because there are few inflammatory cells on the DRG primary culture. Thus, it is suggested that the upregulation of PACAP induced by DPP-4 inhibitors in the vicinity of DRG contributes significantly to the extension of neurite length among DRG neurons, thereby promoting a neurotrophic response. These findings highlight the complex interplay between various neurotrophic factors and their regulatory pathways, underscoring the need for further investigation into the intricate mechanisms governing neurotrophic effects and their therapeutic implications, particularly in the context of DPN. Inhibiting the activity of DPP-4 in the PNS may lead to potential therapeutic implications for DPN.

There are three limitations in this study. First, we have not verified whether DPP-4 inhibitors and PACAP could effectively restore the elongation of neurite length of DRG neurons in diabetic model animals. It is expected that they promote the neurite length of DRG neurons in diabetic models. While there exists promising literature that indicates the potential of DPP-4 inhibitors to enhance neurite length in DRG neurons within type 1 diabetic rodent models by preventing neuropathy and IENFDs reduction [42,43,44,45], it remains ambiguous whether their efficacy stems solely from their hypoglycemic properties or if they exert a direct influence on the PNS. This uncertainty underscores the need for further research to delineate the specific mechanisms underlying their impact on neuronal morphology within diabetic contexts. Clarifying these mechanisms could not only enhance our understanding of DPN but also potentially inform the development of more targeted therapeutic interventions aimed at mitigating DPN. It is crucial to also assess the neuroprotective effects of PACAP administration in vivo. If PACAP exerts neuroprotective effects on DRG neurons in vivo, further studies, including gene expression analysis in DRG neurons, could provide new insights into DPN management. Second, the exploration of PACAP signal transduction in DRG neurons remains an uncharted territory that warrants detailed investigation into its molecular mechanisms. Our previous studies have highlighted the neuroprotective and neurite outgrowth-promoting effects of glucagon and GLP-1 analogs, along with the upregulation of PKA activity, using the 50B11 cells [47,48]. In this study, our MTT assay revealed that DPP-4 inhibitors and PACAP enhanced cell viability against H_2_O_2_. Building upon this foundation, the scope extends to evaluating the impacts of DPP-4 inhibitors and PACAP in the 50B11 cells. This comprehensive approach promises a deeper understanding of the signaling pathways and regulatory mechanisms governing neuronal responses, thereby paving the way for targeted interventions and therapeutic strategies aimed at mitigating neurological disorders and enhancing neuronal function. Third, assessing the effects of PACAP administration on peripheral polyneuropathy in vivo presents a considerable challenge due to the ubiquitous expression of PACAP receptors, notably the PAC1 receptor, across various tissues, which can lead to systemic effects irrespective of central or peripheral localization [61,62,63,64]. Complicating matters further, PACAP can penetrate the blood-brain barrier via transporters, potentially influencing the central nervous system, thereby confounding efforts to isolate its effects on peripheral neuropathy [65]. This multifaceted nature of PACAP as a sensory transmitter projecting to the spinal cord adds another layer of complexity to the evaluation process [53]. Addressing this complexity demands the development of PNS-specific PACAP or PAC1 knockout (KO) diabetic animal models for more precise analysis of DPN. Additionally, exploring DPN using PNS-specific DPP-4 KO diabetic animal models presents an intriguing avenue for research in this field. Such advancements in animal models will be crucial for gaining a deeper understanding of the mechanisms underlying PACAP’s effects on DPN and, ultimately, for developing targeted therapeutic interventions.

In conclusion, the groundbreaking findings of the current study have unveiled a significant revelation regarding the influence of DPP-4 inhibitors and PACAP, a substrate of DPP-4, on the promotion of neurite outgrowth in DRG neurons, thereby shedding new light on potential therapeutic avenues for peripheral neuropathy. Notably, this study marks the first documented instance where DPP-4 inhibitors have been shown to directly facilitate the elongation of neurites in DRG neurons, marking a pivotal milestone in our understanding of their neurotrophic effects. Furthermore, the established correlation between DPP-4 inhibitors and PACAP among DPP-4 substrates further underscores the intricate interplay within these neurotrophic pathways, presenting a compelling framework for future research and clinical interventions aimed at addressing neuropathic conditions. These results collectively offer a paradigm-shifting perspective and hold promising implications for advancing treatment strategies in peripheral neuropathy management.

## 4. Materials and Methods

### 4.1. Animals

For the purposes of this research study, male C57BL/6J (BL6) mice (Japan SLC, Hamamatsu, Japan) were utilized as the experimental subjects. These mice were housed in groups of 3–5 per cage under controlled environmental conditions, maintaining a temperature of 22 ± 2 °C and following a 12-h dark-light cycle, ensuring their welfare with unrestricted access to food and water throughout the study duration. The euthanasia of mice was performed by administering an overdose of anesthesia. The ethical framework for this experiment was established and overseen by the Aichi Medical University Institutional Animal Care and Use Committee, with approval granted under Code 2023-69 on 10 July 2023. All procedures were performed in accordance with the guidelines of the Aichi Medical University Institutional Animal Care and Use Committee and followed the “3R”, “Replacement of animals by alternatives wherever possible”, “Reduction in the number of animals used”, and “Refinement of experimental conditions and procedures to minimize the harm to animals” with the guidelines outlined in the UK Animals (Scientific Procedures) Act 1986, revised in 2012.

### 4.2. Primary Culture of Dorsal Root Ganglion (DRG) Neurons and Evaluation of Neurite Outgrowth

DRG neurons were meticulously prepared from 4-week-old male BL6 mice (*n* = 20) according to established protocols [51], involving a rigorous process beginning with the deep anesthesia of the animals using a cocktail comprising medetomidine hydrochloride (75 μg/mL; Domitol; Meiji Seika Pharma, Tokyo, Japan), midazolam (400 μg/mL; Dormicum; Maruishi Pharm, Osaka, Japan), and butorphanol (500 μg/mL; Vetorphale; Meiji Seika Pharma) delivered intraperitoneally at a precise dosage of 1 mL/kg. Following anesthesia, DRGs were carefully collected and subjected to dissociation using 0.42% collagenase (Fujifilm, Tokyo, Japan), triturated through a series of heat-polished glass pipettes, and diluted in a meticulously crafted medium consisting of Dulbecco’s Modified Eagle Medium with 5.5 mM glucose (Fujifilm) supplemented with insulin-transferrin-selenium (Life Technologies, Carlsbad, CA, USA) and maintained at a constant temperature of 37 °C for a duration of 30 min. Subsequently, the isolated DRG neurons were delicately seeded onto glass coverslips that had been meticulously coated with 0.1% poly-L-lysine (Sigma-Aldrich Japan, Tokyo, Japan) in Phosphate-Buffered Saline (PBS, Fujifilm). These cultured DRG neurons were then subjected to various treatments involving Diprotin A (Fujifilm), sitagliptin phosphate monohydrate (BIOSYNTH, Staad, Switzerland), linagliptin (Funakoshi, Tokyo, Japan), PACAP (Fujifilm), NPY (Fujifilm), and SDF-1a (Fujifilm) under precise incubation conditions at 37 °C, forming the basis of extensive experimental investigations aimed at elucidating critical neurobiological phenomena.

After a period of 24 to 48 h of culture at 37 °C, DRG neurons underwent fixation using 4% paraformaldehyde (PFA; Fujifilm) in PBS for 30 min at room temperature (RT). Following fixation, the DRG neurons were subjected to blocking with 1% bovine serum albumin (BSA; Fujifilm) in PBS for another 30 min at RT. Subsequently, immunostaining was performed using a rabbit polyclonal Anti-Neurofilament H antibody, clone NE14, and Alexa Fluor^®^ 488 Conjugated anti-neurofilament light-chain antibody (diluted at 1:1000; MAB5256X, Merck Millipore, Darmstadt, Germany) overnight at 4 °C. Following five consecutive 5-min washes with PBS at RT, coverslips were counterstained with diaminidophenylindole (DAPI; Merck Millipore). The resulting images were captured using a charge-coupled device camera (DP70, Olympus Optical, Tokyo, Japan) mounted on a fluorescence microscope (IX73, Olympus Optical). Neurite outgrowth was assessed by observing 10 to 20 neurons per coverslip and analyzing the images with ImageJ software version 1.53v (National Institutes of Health, Bethesda, MD, USA).

### 4.3. DPP-4 mRNA Expression in DRG and Sciatic Nerves

DRGs, sciatic nerves, livers, hearts, and skeletal muscles were collected from anesthetized 4-week-old male BL6 mice (*n* = 3). For anesthesia, the same cocktail, as mentioned previously, was used for the animals. RNAs were meticulously extracted from a diverse array of biological samples, encompassing frozen specimens of DRGs, sciatic nerves, livers, hearts, and skeletal muscles, employing the renowned Isogen extraction kit (Nippon Gene, Toyama, Japan), ensuring utmost precision and reliability. The quantification of RNA content was meticulously conducted through spectrophotometric analysis, guaranteeing accurate measurements. Subsequently, commencing with 1 milligram of RNA, the synthesis of complementary DNA (cDNA) was expertly executed utilizing the ReverTra Ace kit (TOYOBO, Osaka, Japan), following a meticulously calibrated protocol: Step 1 entailed incubation at 37 °C for 15 min, Step 2 at 50 °C for 5 min, and Step 3 at 98 °C for 5 min, ensuring optimal cDNA yield and quality. The cDNA obtained was then subjected to RT-PCR using the highly efficient THUNDERBIRD SYBR qPCR Mix (TOYOBO), wherein a precisely orchestrated protocol was followed: Step 1 initiated at 95 °C for 30 s, Step 2 at 95 °C for 15 s, 60 °C for 30 s, and 72 °C for 30 s (repeated for 40 cycles), and Step 3 included a melting phase at 95 °C for 15 s, 65 °C for 60 s, and 95 °C for 15 s (conducted once). Detailed information regarding the primer sequences utilized for DPP-4 amplification includes the Forward primer: 5′- TGTGGATAGCAAGCGAGTTG-3′ and Reverse primer: 5′-CACAGCTATTCCGCACTTGA, resulting in a PCR product of 106 base pairs. The ensuing PCR products were meticulously analyzed through electrophoresis using a 2% agarose gel (TaKaRa Bio, Shiga, Japan) and were visualized for DPP-4 expression employing a state-of-the-art gel imager (FAS-V; Nippon Genetics, Tokyo, Japan), ensuring precise detection and analysis of genetic markers.

### 4.4. Immunocytochemistry and Frozen Section Staining

DRGs were meticulously collected from anesthetized 4-week-old male BL6 mice (*n* = 3) according to established protocols [38], following transcardial perfusion with a solution containing 4% PFA in PBS to ensure proper fixation of the DRGs. For anesthesia, the same cocktail, as mentioned previously, was used for the animals. Subsequently, the collected DRGs underwent post-fixation with 4% PFA in PBS, followed by cryopreservation overnight in a solution of 10% sucrose (Fujifilm) in PBS. This was succeeded by gradual transition and maintenance in increasing concentrations of sucrose solutions: first in 20% sucrose in PBS and then in 30% sucrose in PBS to facilitate optimal tissue preservation. The fixed DRGs were then precisely sectioned at a thickness of 5 μm using a cryostat (Leica Biosystems, Tokyo, Japan). To enhance permeability, the sections were treated with 0.05% Triton-X100 (Sigma-Aldrich Japan) in PBS at room temperature for 30 min and subsequently blocked with 1% BSA in PBS for 30 min at room temperature. Next, primary antibodies, such as the species polyclonal anti–DPP-4 antibody (1:200; Santa Cruz Biotechnology, Inc., Dallas, TX, USA), were applied to the glass coverslips and incubated overnight at 4 °C. Following rigorous washing steps with 0.05% Triton-X100 in PBS three times for 5 min each at room temperature, secondary antibodies such as Alexa Fluor 594–coupled goat anti-species antibody (1:200; Invitrogen, Waltham, MO, USA) were applied and left to incubate for 1 h at room temperature in a dark box to avoid photobleaching. Further washing with PBS five times for 5 min each at room temperature ensured the removal of unbound antibodies. Finally, coverslips and tissues were counterstained with DAPI, and high-resolution images were captured using a charge-coupled device camera (DP70, Olympus Optical, Tokyo, Japan) mounted on a fluorescence microscope (IX73, Olympus Optical) to enable detailed analysis and visualization of the DRG structures.

### 4.5. DPP-4 Activity

DRGs and livers were collected from anesthetized 11-week-old male BL6 mice (*n* = 4). For anesthesia, the same cocktail as described above was used for the animals. DPP-4 activity in the lysates of DRGs and livers was measured by using a DPP-4 activity assay kit (MAK088, Sigma-Aldrich Japan) according to the manufacturer’s instructions. The values were normalized to the total protein levels assessed with a bicinchoninic acid protein assay (Fujifilm).

### 4.6. MTT Assay

For the MTT assay, 50B11 cells were seeded into 24-well plates at a density of 5 × 10^4^ cells/well. These cultured 50B11 cells were then subjected to various treatments involving 500 μM Diprotin A, 250 μM sitagliptin phosphate monohydrate, 10 μM linagliptin, 1 μM PACAP under precise incubation conditions at 37 °C. After 24 h, oxidative stress was induced by 0.1mM H_2_O_2_ (Fujifilm). After a period of 24 h of culture at 37 °C, 50B11 cells were subjected to MTT (Dojindo, Kumamoto, Japan) for 3 h at 37 °C. Therefore, each MTT inner salt was dissolved in dimethyl sulfoxide for 30 min at room temperature (RT). Then, the absorbance of 100 μL of solutions at 530 nm was measured on a microplate reader (VersaMax, Molecular Devices, Sunnyvale, CA, USA). Cell viability was calculated using the following formula: Cell viability (%) = 100 × sample OD/H_2_O_2_-untreated control OD (OD: optical density). Each OD value was calculated by subtracting the background value from each absorbance value.

### 4.7. Statistical Analysis

The analysis of two groups was conducted using a Student’s *t*-test, and the comprehensive analysis of the entire group was conducted using a one-way analysis of variance, incorporating the Bonferroni correction to account for multiple comparisons, with group values meticulously presented as mean ± standard deviation (SD). The threshold denoting statistical significance was set at a *p*-value of less than 0.05, ensuring robust and reliable results. Notably, the experimental design obviated the need for randomization of mice, thereby maintaining consistency and minimizing potential confounders. Moreover, to uphold the integrity of the analyses, all procedures were carried out by personnel who remained blind to the identities of the animals, thereby reducing the risk of bias and ensuring the scientific rigor of the study.

## Data Availability

Data is contained within the article.
